# Formulation of Genetic Counseling Format for Adult Bangladeshi Patients with Acute Myeloid Leukemia 

**DOI:** 10.1155/2018/1534090

**Published:** 2018-04-29

**Authors:** M. Z. Rahman, L. Nishat, Z. A. Yesmin, L. A. Banu

**Affiliations:** Department of Anatomy, Bangabandhu Sheikh Mujib Medical University (BSMMU), Dhaka, Bangladesh

## Abstract

With the advancement of medical genetics, particular emphasis is given on the genetic counseling worldwide. In Bangladesh, genetic counseling services are not yet developed. Acute myeloid leukemia (AML) is a malignant disease of the myeloid cells of bone marrow. Like other malignant diseases, it may result from a mutation in the DNA. A genetic counseling format will educate the AML patients and provide appropriate medical and emotional support. The aim of this descriptive cross-sectional study was to develop a genetic counseling format for adult Bangladeshi patients with AML. Taking this into account, a draft format was prepared by reviewing relevant documents available online which was later analyzed by an expert panel through a group discussion and thus a proposed format was developed. To make the format effective in the perspective of Bangladeshi population, the proposed format was applied in counseling, and thus a final format was developed in the English language. This format will educate the counselors, clinicians, and patients about the utility and importance of the genetic counseling and genetic tests. Also, the patients feel comfort regarding the whole counseling process and going for postcounseling treatments and advice. Though it is written in English, it may be translated into mother tongue for better communication during counseling.

## 1. Introduction

Acute myeloid leukemia (AML) is a malignant disease of the myeloid cells of bone marrow in which precursors of blood cells are arrested in an early stage of development [1]. The synonyms of AML are acute myelogenous leukemia and acute nonlymphocytic leukemia [2]. According to WHO, Bangladesh is experiencing increasing cancer burden with an estimated 122,715 new cancer cases in 2012. In case of Bangladesh, AML was the most frequent hematological malignancy at the rate of 28.3% [3]. Survival with AML depends on age [4]. The incidence of therapy related (secondary) AML is 10–20% of all AML cases [5]. According to Hossain et al. [3] the median age at onset of AML in Bangladesh is 35 years which is an active age for society. Untreated AML cases are typically fatal within a short period of time [6]. The chance of an individual developing cancer depends on both genetic and nongenetic factors. AML “is a genetic disease” [7] and, like other cancers, it may results from mutations in the DNA. Familial AML is a rare type of inherited leukemia which is transmitted by a nonsex chromosome in a dominant fashion. Several genes have been identified with their incidence, treatment, and prognostic outcomes. In spite of the rapid development of genetic techniques for diagnosis, genetic disorders remain undetected for several years [8]. So, there are some complex issues that need to be communicated with patients. “Genetic counseling is a communicative process” [9]. As genetic testing is a useful tool in the clinical management of disease, the importance of genetic counseling is increasing worldwide with the advancement in the field of genetics. In context of genetic counseling, the aim is to communicate information regarding the personal impact of disease, so that individuals can take decision from the informed choices regarding options for risk management, disease surveillance, and predictive genetic testing and also adapt to the emotional, psychological, medical, social, and economic consequences of the test results with treatment. Thus, genetic counseling enables one to assess the risk of cancer without the use of genetic tests. As a result, genetic counseling acts as an integral part for the cancer risk assessment process [10]. In this context, family history holds important information about an individual's past and future life. For this reason, need is felt to communicate the complex cancer genetic information to the AML patients using everyday language. The workshop Genetic Counseling/Consultations in Southeast Asia at the 10th Asia Pacific Conference on Human Genetics in Kuala Lumpur, Malaysia, in December 2012 aimed at addressing culture and context-specific genetic counseling/consultation practices in Southeast Asia. There are a large number of AML patients in Bangladesh and so far no organized counseling service is available for the patients or their relatives. Responding to the global trends, the human genetics research in Bangabandhu Sheikh Mujib Medical University (BSMMU) has already been modernized with the help of Higher Education Quality Enhancement Project (HEQEP) of University Grants Commission of Bangladesh. It would, therefore, be a great opportunity to modernize our medical knowledge in this field for the betterment of the patient care. The unique aspects of AML genetic information can guide a course of action to minimize distress and maximize benefit for both the patient and the family. This study aims to present formats of practice recommendations for genetic counselors conveying AML genetic counseling with surveillance and management in a manner so that all patients can be provided with the same information by using a structured, valid, and reliable genetic counseling format. So, the purpose of this study was to develop a genetic counseling intake format for AML patients. Presently in Bangladesh, there is no standard genetic counseling format for counseling patients. If a genetic counseling format is available for any disease, then any one from the counseling team can counsel the patients with minimum training and deliver the same information to the patients.

## 2. Methods

The present research is a cross-sectional descriptive study. It was carried out in the Department of Anatomy, BSMMU, and the counseling was done in the Hematology Department of the university. The study was approved by the Institutional Review Board (IRB) of BSMMU.

**Figure 1:**
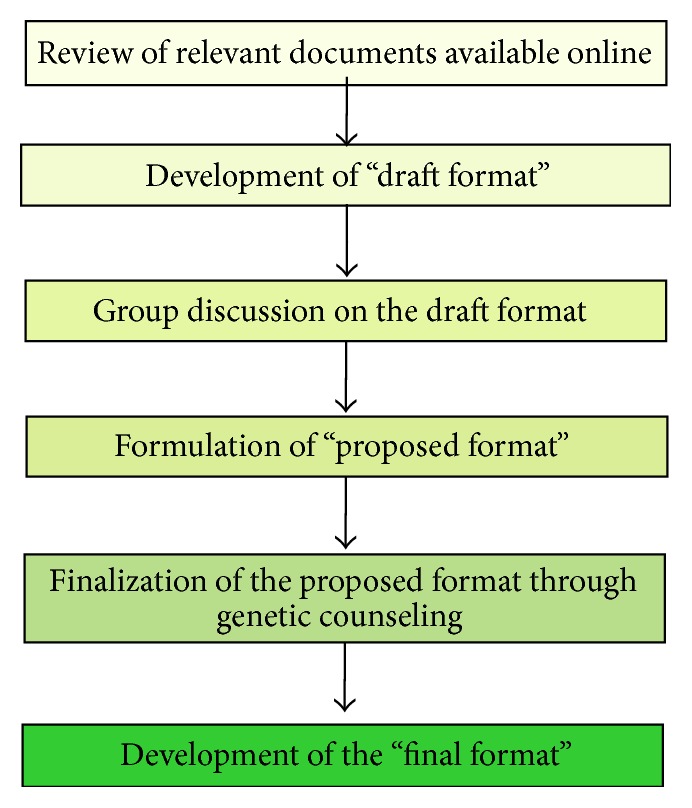
Steps of development of genetic counseling format for AML patients.

The study involves many steps which are shown in Figure 1.

The AML counseling formats available online, other cancer related counseling formats, journal articles, and books related to AML counseling were reviewed and a “draft format” for genetic counseling was developed for the adult Bangladeshi patients with AML. A group discussion for analysis of the draft format was organized. Five specialists (two hematologists, one educationist, one psychiatrist, and one counselor) were selected for the discussion. Relevant documents were sent to the participants seven days prior to the group discussion. The participants provided a written feedback form after the discussion. A “proposed genetic counseling format” was developed for AML patients by modifying the draft format according to the feedback of the group discussion participants. Both the draft and the proposed formats were in English. Genetic counseling of 24 adult Bangladeshi AML patients using the proposed format was conducted by a four-member counseling team. A “final genetic counseling format” was developed both in English and in Bengali (the mother tongue) after counseling.

## 3. Ethical Issue

The study was conducted after receiving ethical approval from the Institutional Review Board (IRB) of BSMMU. All the participants of the group discussion and the patients participated in the genetic counseling voluntarily. A written informed consent form was provided to the participating patients, and counseling was conducted only after getting their consent in that form. Anonymity was strictly maintained for both the patients and the group discussion participants. Confidentiality of the information of the patients was maintained.

## 4. Results and Discussion

The draft format for genetic counseling was prepared by analyzing journal articles related to the AML counseling, counseling formats available online for AML, and other cancers and books. The draft format was constructed on the following parameters:

- Instructional note related to the way of conversation and filling up the form
- General information of the patient
- Present medical history of the patient
- Three generations' family history of the patient and pedigree chart
- Findings of the laboratory investigations of the patient
- Information to be provided by the counselor to the consult
- Summary of the case and card for the consult.

The development of genetic counseling format aims to assist AML patients and genetic counselors in understanding the occurrence, probable cause, and available genetic test with related management of the AML. The format acts as a guideline for the counselor. According to Nishat [11] and Yesmin [12] analysis of available relevant documents is an important step for formulating any new format. Journal articles related to counseling, counseling formats for AML and other cancers available online, and books on genetic counseling were helpful for idea generation and construction of the draft format.

The draft format was presented to the specialists for group discussion. The participants discussed various aspects of the draft format. The opinion of the group discussion participants on the draft format is presented in Table 1.

Group discussion is an effective process in qualitative research [13]. Information related to the disease, educational and psychological aspects, and the counselor's view about the draft format was collected in the group discussion. Necessary modification of the draft format was done according to the findings of the group discussion and the “proposed format” for genetic counseling was prepared. The proposed format contains the following parameters:

- General information
- Personal information
- Medical history
- Environmental factor exposure history
- Family history
- Laboratory investigations
- Summary of the case
- Comment of the genetic counselor.

Family history includes patient's first-degree (50% shared gene), second-degree (25% shared gene), and third-degree (12.5% shared gene) relatives. In case of the pedigree chart, only three generations of the index case were included.

The researchers (the first and third authors), a geneticist, and a nurse conducted the genetic counseling of 24 adult Bengali Bangladeshi patients with AML using the proposed format. Genetic counseling was performed to validate the proposed counseling format. The counselors and the patients experienced few linguistic problems during counseling. The proposed format was in English, but the mother tongue of the patients as well as counselors is Bengali. Payne et al. [14] reported that genetic counseling is a communication process to provide information and support patients so that they could understand the medical facts. On the basis of the counseling feedback, the proposed format was finalized without any major modifications. The psychological orientation of a person is significantly affected by the mother tongue or known language [15]. But it was observed that it is quite difficult to communicate with patients in the English version of the format. So the final format was also translated into Bengali to overcome the linguistic barrier and for better communication with patients during counseling. The English version of the final format is attached herewith (available here).

## 5. Conclusion

The final genetic counseling format will help a variety of professionals to counsel AML patients. The face-to-face description and genetic explanation of AML to each patient make them aware about the genetic background of the disease and sharing the information with the family member and other members of the society.

## Figures and Tables

**Table 1:** Outcome of the group discussion on the draft format.

Area where modifications are required	Modification
*Overall organization of the draft format*	Need to be organized with headings & subheadings
*Parameters*	
Instructional note	Should be omitted
General information	Should be divided into two broad headings:
	(A) General information
	(B) Personal information
	Identification (ID) number, serial number, and emergency contact number should be included in general information
	Birth history, consanguinity of marriage, and history of substance abuse should be included in personal information
Present medical history	Should be divided into two broad headings:
	(A) Medical history and
	(B) Environmental factor exposure history
Family history	Will be arranged according to the degree of relation

## Abbreviations

AML: Acute myeloid leukemia
BSMMU: Bangabandhu Sheikh Mujib Medical University
HEQEP: Higher Education Quality Enhancement Project.

## Supplementary Materials

Supplementary Materials English version of the final format of genetic counseling of adult Bangladeshi patients with AML is included as the supplementary material.
