# Supplementary material for: Formulation of Genetic Counseling Format for Adult Bangladeshi Patients with Acute Myeloid Leukemia
Source: Genet Res Int. 2018 Apr 29;2018:1534090. doi: 10.1155/2018/1534090 (PMC5949166; doi:10.1155/2018/1534090)
Supplement: Supplementary Materials — English version of the final format of genetic counseling of adult Bangladeshi patients with AML is included as the supplementary material. [file 1534090.f1.pdf]

Genetic Counseling Format for Adult Bangladeshi AML Patients

Please use remark or tick mark (√) if necessary

A. General Information

1. ID No. :
2. Patient Name :
3. Age in year :
4. Sex : ☐ Male ☐ Female ☐ Other
5. Marital status : ☐ Single ☐ Married ☐ Divorced ☐ Widowed ☐ Separated
6. Number of children : _____boy(s) ____girl(s)
7. Educational status :
8. Occupation :
9. Address
   - Permanent :
   - Present :
   - Cell phone :
   - E-mail :
10. Please mention who referred you for genetic counseling : ☐ Physician ☐ Patient ☐ Family member ☐ Self ☐ Other

B. Personal Information

1. Birth history : Full term- ☐ Yes ☐ No
   Delivery- ☐ Vaginal ☐ C-section

2. Consanguinity of marriage : ☐ Yes ☐ No

3. Female patient
   - i) Do you have menstrual periods? : ☐ Yes ☐ No
     - If yes, then your periods : ☐ Regular ☐ Irregular ☐ Painful
     - Age at start of menstrual period :
     - How often do they occur? Every____ days
     - How long do they last? : ____ days
     - If no, when it stop? :
     - After that if any Vaginal bleeding, discharge :
   - ii) Have you ever been pregnant? : ☐ Yes ☐ No
     if yes, then fill the following-
     Total number of pregnancies________
     Total number of miscarriages/ abortions_____
     Total number of living children ________

4. History of substance use
   - i) Did you use ever tobacco? : ☐ Yes ☐ No
     If yes, then
     ☐ Cigarette ☐ Chew ☐ Snuff
     If current smoker, then ____________ per day
   - ii) Do you drink alcohol? : ☐ Yes ☐ No
     If yes, then mention detail _______________
   - iii) Have you ever shared needles? : ☐ Yes ☐ No
     If yes, then mention detail (medication/addiction purpose)

C.	**Medical History**		
1.	When have your disease first diagnosed?	:	________age in year
2.	Which of the following symptoms before diagnosis of the present disease you were going to the doctor	:	☐ Fever ☐ Weakness ☐ Headache ☐ Night sweats ☐ Shortness of breath<br>☐ Loss of appetite ☐ Swelling in the abdomen ☐ Bleeding gum<br>☐ Weight loss ☐ Frequent nose bleeding ☐ Excess bruising ☐ Bone/joint pain
3.	Before diagnosis of AML, have you taken doctor advice for any other blood disease?	:	☐ Yes ☐ No
4.	Do you any birth disease (congenital) diagnosed previously?	:	☐ Yes ☐ No
5.	Have you had any other cancer?	:	☐ Yes ☐ No
6.	When you were born, how old was your father and mother?	:	
7.	Do you have any big brother or sister?	:	☐ Yes ☐ No<br>If yes, then how much age difference from your immediate big brother/sister?
8.	Did you ever take chemotherapy/radiotherapy for treatment purpose?	:	☐ Yes ☐ No
9.	Did you frequently encounter industrial or medical science radiation (X-ray, CT scan)?	:	☐ Yes ☐ No

D.	**Environmental Factor Exposure History**		
1.	Have you ever exposed to over/unusual smoke in your life?	:	☐ Yes ☐ No<br>If yes, then<br>☐ Motor vehicle ☐ Industrial ☐ Formalin ☐ Insecticide ☐ Others
2.	Have you ever worked in the industries/factories?	:	☐ Yes ☐ No<br>If yes, then<br>☐ Cement factory ☐ Coal mine ☐ Fertilizer factory ☐ Petroleum refining<br>☐ Paint factory ☐ Paper factory ☐ Rubber factory ☐ Shoe factory<br>☐ Others

E. Family History

Take 3 generations of the family history to draw the pedigree to identify the inheritance patterns

Pedigree chart [Follow the instruction]

Family Origin ________________________________

I

II

III

F. Laboratory investigation

Blood examination finding :
Bone marrow examination finding :
CSF marrow examination finding :
Immunophenotype examination finding :
Genomic examination finding :
Karyotype :
FISH :
PCR :
Molecular analyses :

G. Summary of the case

H. Comments of the genetic counselor

Types of AML	:	□ Sporadic □ Familial □ Hereditary
Inheritance pattern	:	□ Autosomal Dominant/ Recessive<br>□ X-linked Dominant/ Recessive<br>□ Mitochondrial
Genetic test:	:	□ Done □ Desire to be done □ Not desire to be done

Name of Genetic counselor with Seal

Address of Genetic counselor

Place of Genetic counseling

Card for Consultand

Institution name: ____

Name of Consultand: _______________________________________ Age in Year________

Types of AML:

□ Sporadic

□ Familial

□ Hereditary

Inheritance pattern:

□ Autosomal Dominant/ Recessive

□ X-linked Dominant/ Recessive

□ Mitochondrial

Genetic test: □ Done □ Desire to be done □ Not desire to be done

Suggested genetic Test: ____

Follow up (provable date and time):

Genetic counseling Summary: ____
